# The Heterochromatin Block That Functions as a Rod Cell Microlens in Owl Monkeys Formed within a 15-Myr Time Span

**DOI:** 10.1093/gbe/evab021

**Published:** 2021-02-03

**Authors:** Hideyuki Tanabe, Ken Takeshi Kusakabe, Hiroyuki Imai, Shin-Ichi Yokota, Takeshi Kuraishi, Shosaku Hattori, Chieko Kai, Akihiko Koga

**Affiliations:** 1 Department of Evolutionary Studies of Biosystems, The Graduate University for Advanced Studies, SOKENDAI, Hayama, Japan; 2 Laboratory of Veterinary Anatomy, Joint Faculty of Veterinary Medicine, Yamaguchi University, Japan; 3 Amami Laboratory of Injurious Animals, Institute of Medical Science, The University of Tokyo, Kagoshima, Japan; 4 Institute of Industrial Science, The University of Tokyo, Komaba, Japan; 5 Primate Research Institute, Kyoto University, Inuyama, Japan

**Keywords:** adaptation, nocturnality, satellite DNA, primate, mammal

## Abstract

In rod cells of many nocturnal mammals, heterochromatin localizes to the central region of the nucleus and serves as a lens to send light efficiently to the photoreceptor region. The genus *Aotus* (owl monkeys) is commonly considered to have undergone a shift from diurnal to nocturnal lifestyle. We recently demonstrated that rod cells of the *Aotus* species *Aotus azarae* possess a heterochromatin block at the center of its nucleus. The purpose of the present study was to estimate the time span in which the formation of the heterochromatin block took place. We performed three-dimensional hybridization analysis of the rod cell of another species, *Aotus lemurinus.* This analysis revealed the presence of a heterochromatin block that consisted of the same DNA components as those in *A. azarae*. These results indicate that the formation was complete at or before the separation of the two species. Based on the commonly accepted evolutionary history of New World monkeys and specifically of owl monkeys, the time span for the entire formation process was estimated to be 15 Myr at most.

**Significance**

Many nocturnal mammals have a microlens structure in their photoreceptor cells that allows improved night vision. Owl monkeys underwent a shift from diurnal to nocturnal lifestyle relatively recently and acquired this structure. Our analysis of the owl monkey retina showed that the time span for the entire acquisition process was 15 Myr or less. This estimate could be obtained by examining owl monkeys; other nocturnal mammals may have acquired the structure in more remote times or may have been nocturnal ever since early mammals.

## Introduction: Rod Nucleus of Owl Monkey

In photoreceptor cells of vertebrate eyes, incoming light passes through the nucleus region and then reaches the photoreceptor region, where opsin proteins reside. Rod cells are the photoreceptor cell that have a higher reactivity to weak light than other types of photoreceptor cells. In rod cells of many nocturnal mammals, heterochromatin localizes to the central region of the nucleus and serves as a lens to send light efficiently to the photoreceptor region (Solovei et al. 2009, 2013). In general, heterochromatin is distributed in the nuclear periphery due to its connection to the nuclear lamina, and the inner space of the nucleus is occupied by euchromatin. This positioning pattern is called the “conventional nuclear architecture,” and the exceptional pattern observed in rod cells of nocturnal mammals is called the “inverted nuclear architecture” (Solovei et al. 2009). Simian primates (infraorder Simiiformes), which include humans, apes, macaques, baboons, and New World monkeys, are characterized by clear diurnal activity. However, there is one exception: owl monkeys (genus *Aotus*). Owl monkeys, also called night monkeys, are commonly thought to have undergone a shift from diurnal to nocturnal lifestyle. The spatial distribution of euchromatin and heterochromatin in the rod nucleus was examined in various primates (Joffe et al. 2014), and an owl monkey (*Aotus* sp. in their description) was shown to have a heterochromatin block at the center of the nucleus. The other simian primates examined, including human, macaque, marmoset, and capuchin, exhibited the conventional nuclear architecture. Although a heterochromaton block was observed, the owl monkey rod cell did not show the complete inverted nuclear architecture, as heterochromatin was also distributed in the outer regions of the heterochromatin block. However, as subsequently documented in our research using *Aotus azarae* (Koga et al. 2017) and explained below, the heterochromatin residing outside differs in DNA sequence from those found in the heterochromatin block. Thus, at least in *A. azarae*, the formation of the heterochromatin block is complete even if the inverted nuclear architecture is incomplete.

## Components of Heterochromatin Block

When the heterochromatin block was first found in an owl monkey (Joffe et al. 2014), the type of heterochromatin in the block remained an open question. We previously cloned three types of megasatellite DNAs that exist in the genome of *A. azarae* (Prakhongcheep, Hirai, et al. 2013; Prakhongcheep, Chaiprasertsri, et al. 2013). The three megasatellite DNAs consisted of repeat units of 185, 344, and 187 bp, and were named OwlAlp1, OwlAlp2, and OwlRep, respectively. Of these, OwlRep was unique to owl monkeys; satellite DNA similar in nucleotide sequence to OwlRep was not found in the other New World monkeys or more distantly related primates that we examined (Koga et al. 2017; Nishihara et al. 2018). Using the three megasatellite DNAs as probes for different colors, we conducted a three-dimensional fluorescent in situ hybridization (3D-FISH) analysis of rod cells from *A. azarae* (Koga et al. 2017). This analysis revealed that the heterochromatin block comprises OwlRep and OwlAlp2 as the main and subsidiary components, respectively. OwlAlp1 was distributed in the outside region of the heterochromatin block and was shown to act as centromeric repetitive DNA (Prakhongcheep, Hirai, et al. 2013; Oizumi et al. 2019).

## Question

**Fig. 1:**
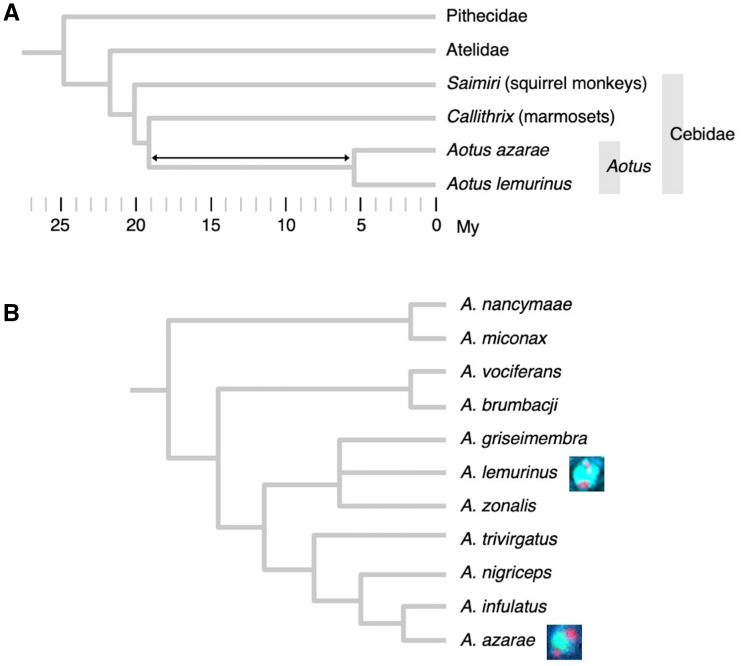
Phylogenetic relationship of New World monkeys. (*A*) The two focal *Aotus* species in the present study and other key taxa of New World monkeys. The phylogeny of the species and taxa shown in this panel is widely accepted as illustrated here. Perelman et al. (2011) estimated divergence times of various primates using nucleotide sequence data of 54 nuclear gene regions. Their estimates were reflected in the illustration in this panel. The double-headed arrow shows the maximum time span in which the microlens formation took place (see text for details). (*B*) Species in genus *Aotus*. A clear consensus has not been reached on the species taxonomy of *Aotus*, including the classification as species or subspecies. This panel shows a classification and taxonomy proposed by Menezes et al. (2010). Note that the branch length does not reflect the time length or degree of variation. The two focal species in the present study are marked with pictures of their microlens structures.

In the present study, we raised a question: How long was the time span in which the entire formation process took place? The species analyzed in our previous study was *A. azarae*. To answer the question, we performed a 3D-FISH analysis of rod cells from *Aotus lemurinus*. In the taxonomy of *Aotus* species, *A. lemurinus* is located not extremely close to *A. azarae* (fig. 1). If the heterochromatin block is also present in *A. lemurinus* and it is assembled from the same components and in the same manner, the formation process can be thought to have been complete at or before the divergence of the two lineages.

### 3D-FISH Analysis

**Fig. 2:**
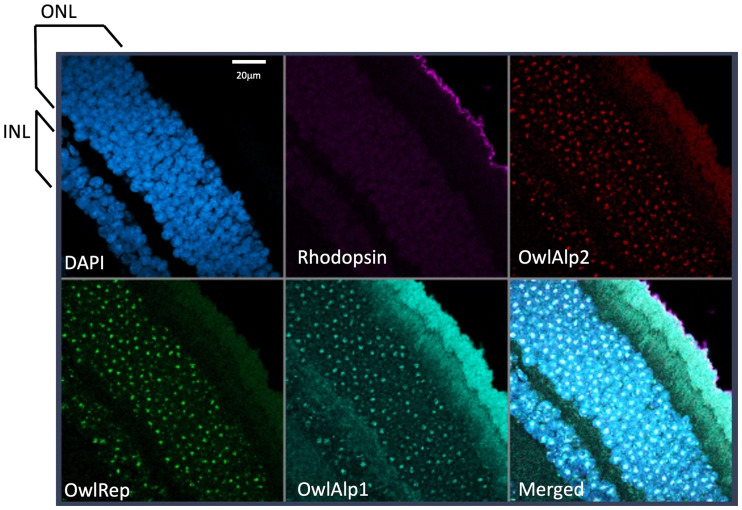
Overview of a tissue slice after 3D-FISH with three megasatellite DNA probes. A representative tissue slice derived from the *A. lemurinus* sample after 3D-FISH with the three megasatellite DNAs combined with immunostaining for rhodopsin and DAPI counterstaining is shown. The DNA probes for OwlAlp1, OwlAlp2, and OwlRep, as well as rhodopsin and DAPI, are represented in different colors: aqua, red, green, purple, and blue, respectively. Rhodopsin signals appear at the upper right side. The image shows a single scanned slice from the image stacks obtained with an LSM980 confocal microscope. INL and ONL indicate the inner nuclear layer and outer nuclear layer, respectively. The white bar in the DAPI image indicates a scale of 20 μm.

**Fig. 3:**
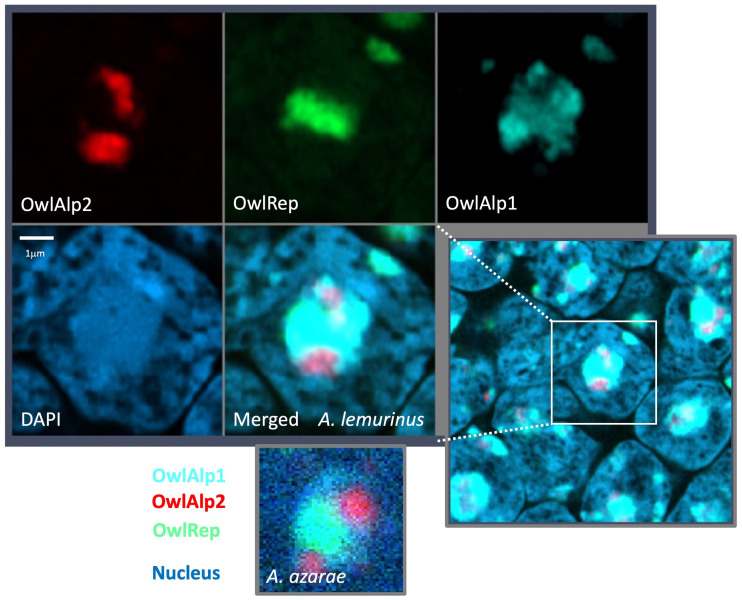
Visualization of three megasatellite DNAs in one slice with high resolution. A representative slice scanned using the Airyscan 2 multiplex mode from LSM980 is shown. A region including multiple nuclei is shown in the lower right panel, and a single nucleus, cut at about the center, is enlarged and shown in the other panels. The colors represent the same as in figure 1. The white bar in the DAPI image indicates a scale of 1 μm. A nuclear image obtained from *A. azarae* in our previous study is shown at the bottom.

**Fig. 4:**
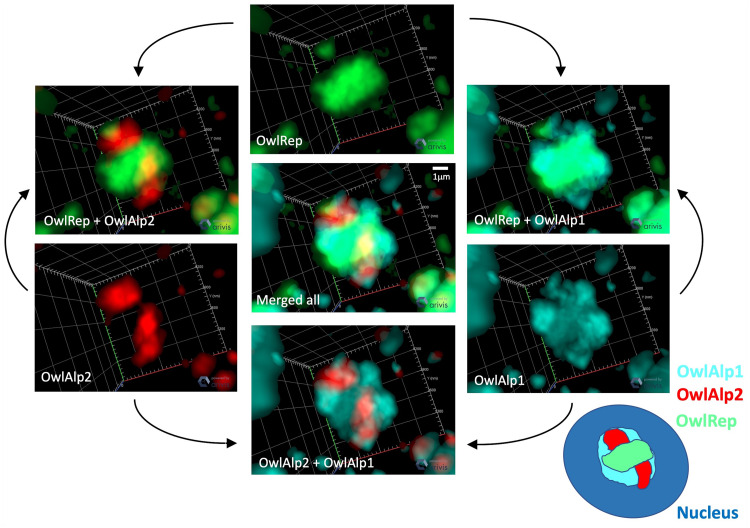
3D visualization of three megasatellite DNAs within a single nucleus. Spatial distribution of three megasatellite DNAs, OwlAlp1, OwlAlp2, and OwlRep, within a typical single nucleus for 3D visualization by reconstruction from image stacks using ZEN 2.3 lite software is shown. Each single-color image of OwlAlp1, OwlAlp2, or OwlRep has been merged with each other showing two or three colors, respectively. The white bar in the “Merged all” image indicates a scale of 1 μm. The overall composition of the nucleus center with the three megasatellite DNAs is schematically illustrated in the lower right part of the figure.

We prepared tissue sections of the retina obtained from an adult of *A. lemurinus* and 3D-FISH combined with immune detection was performed. The retina sample was immunostained with the rhodopsin antibody after 3D-FISH with DNA probes and stained with DAPI for nuclear visualization. For comparative analysis of *A. azarae* and *A. lemurinus*, we used OwlAlp1, OwlAlp2, and OwlRep as DNA probes labeled with three fluorochromes that were hybridized and represented by the same colors as previously shown in *A. azarae* (Koga et al. 2017). Figure 2 shows an overview of tissue slices after 3D-FISH. The images show a representative single scanned slice on the peripheral region of the retina, which consists of the great majority of rod photoreceptor cells. Rhodopsin signals appeared at the upper right side, indicating the outer side of the retina. There were two nuclear layers from the rhodopsin signals; the outer and inner nuclear layers, respectively, containing numerous cell nuclei with positive signals for OwlAlp1, OwlAlp2, and OwlRep. We performed high-resolution analyses of the outer nuclear layer, which consists of nuclei of photoreceptor cells. Using the same methods as those for *A. azarae* (figures S1 and S2 of Koga et al. 2017), multiple cross sections were scanned along the *z*-axis, and the 2D images collected were assembled into a 3D image. In our previous study about *A. azarae*, the great majority of the cells exhibited essentially the same structure. This was also the case in the present study on *A. lemurinus*. Figure 3 shows one of the cross-section images. A region including multiple nuclei is shown in the lower right panel, and a single nucleus, cut at about the center, is enlarged and shown in the other panels. Figure 4 shows a typical single nucleus with 3D visualization, where a heterochromatin block occupying the majority of the nucleus can be observed, suggesting that the heterochromatin block was composed of all three megasatellite DNAs. Together, all image data (figs. 3 and 4) demonstrated that OwlRep (green) occupies a single central space with a slightly rugged surface, connecting to OwlAlp1 (aqua) and OwlAlp2 (red) with different associated surface sides, respectively. OwlAlp2 (red) formed clumps in separate regions, mostly two major and small ones. OwlAlp1 (aqua) also formed clumps, but these were smaller and divided into multiple ones that covered the outer regions surrounding the space occupied by OwlRep (green) and OwlAlp2 (red). As revealed in the two-color merged images (fig. 4), OwlRep and OwlAlp1 were clustered and constituted mainly the backbone of the nuclear lens, with an organization similar to that observed in *A. azarae* (Koga et al. 2017). OwlAlp2 was located in the hollows of the surface of the OwlAlp1 and OwlRep backbone cluster, and rarely overlapped with OwlRep. Thus, we confirmed that the heterochromatin block, which had previously been observed in *A. azarae* rod cells, is also present in *A. lemurinus* and is assembled from the same components and in the same manner.

## Answer

The genus *Aotus* is included in the family Cebidae, together with genus *Callithrix* (marmosets). In our previous study to analyze the *A. azarae* rod nucleus (Koga et al. 2017), we also examined the rod nucleus of *Callithrix jacchus* (common marmoset) for comparison. A heterochromatin block was not observed in this diurnal species. The time point of the divergence between *Aotus* and *Callithrix* has been estimated to be 19.95 Ma (Perelman et al. 2011). Therefore, the formation of the heterochromatin block started then (the left end of the double-headed arrow in fig. 1) or later. To draw information concerning the end point of the formation process, we compared the spatial organization of the three megasatellite DNAs between *A. azarae* and *A. lemurinus*. No obvious differences were observed even with the Airyscan mode of high-resolution scales. These results indicate that the heterochromatin block was established in a common ancestor of these two species and has been maintained until now. Therefore, the minimum (most recent) estimate of the end point is given by the time point of the divergence of the two species (the right end of the double-headed arrow in fig. 1). This has been estimated to be 5.53 Ma (Perelman et al. 2011). The span between these two time points is 14.42 (19.95 − 5.53) Myr. Since an overestimate better fits our purpose than an underestimate, we finally used 15 Myr instead of 14.42 Myr. We conclude that the time span for the entire formation process was 15 Myr or less.

### Ethics

This study did not include any animal experiments; the retina sample used in this study was a surplus of that used in our previous study on retinal morphology (Kuniyoshi et al. 2018), which had been conducted as an approved animal experiment described therein. This study involved a recombinant DNA experiment and was approved in advance by the Recombinant DNA Experiment Safety Committee of Kyoto University (approval number 190058).

### Animal and Retina Sample

The retina sample and the *A. lemurinus* animal from which the sample originated are described in our previous paper (Kuniyoshi et al. 2018). Essential information is as follows: animal identification number, Y20802; sex, male; age, 6 years; sampling process, excision of eyeball from euthanized animal, and isolation of retina.

### 3D-FISH Analysis

To achieve an accurate comparison with our previous study using the *A. azarae* retina sample (Koga et al. 2017), we applied the same methods to the *A. lemurinus* sample, except for a few slight modifications to the pretreatment steps. These modifications were necessary because of the difference in the duration of sample storage. In the previous study, the retina tissue was used immediately after isolation from the animal. The sample used in the present study was a surplus of a tissue sample that was collected for another study 5 years before the present study. To ensure the reproducibility of the methods, we describe the entire process in the Supplementary Material online.

## Supplementary Material

Supplementary data are available at *Genome Biology and Evolution* online.

## Supplementary Material

evab021_Supplementary_Data
