# Supplementary material for: The Heterochromatin Block That Functions as a Rod Cell Microlens in Owl Monkeys Formed within a 15-Myr Time Span
Source: Genome Biol Evol. 2021 Feb 3;13(3):evab021. doi: 10.1093/gbe/evab021 (PMC7991628; doi:10.1093/gbe/evab021)
Supplement: evab021_Supplementary_Data [file evab021_supplementary_data.pdf]

Supplementary Material

The entire process of the 3D-FISH analysis

Retina was taken from an eye and fixed in paraformaldehyde for two h at 4 °C. A part of the sample was used for the initial purpose and the rest was stored in formalin at room temperature. The sample that was stored for five years was washed three times with PBS, sliced to a thickness of 20 µm, placed on glass slides, and frozen until use. The slides were thawed and treated with 0.5% Triton X-100 in PBS for 20 min, followed by washing in PBS. Then, the slides were treated with 0.1N HCl for 10 min and subsequently exposed to 0.004% pepsin in 0.01N HCl at 37 °C for 10 min. Then, the reaction was stopped with 0.05M $MgCl_2$ in PBS for 5 min, followed by a washing step with PBS. Then, the tissue slices were refixed in 1% paraformaldehyde for 10 min, washed in PBS, rinsed in 2×SSC, and soaked in a 50% formamide solution in 2×SSC for at least 12 hours. The probe DNAs for OwlAlp1, OwlAlp2, and OwlRep were fluorescently labeled by nick translation with SpectrumAqua-dUTP, SpectrumOrange-dUTP, and SpectrumGreen-dUTP, respectively. The three labeled DNA probes were mixed, subjected to ethanol precipitation, and resuspended in hybridization solution (50% formamide and 10% dextran sulfate in 2×SSC). The DNA probes were predenatured at 80 °C for 6 min and placed on ice for 1 min. The denatured probes were then applied to fixed tissue slices on the glass slides and sealed under cover glasses with Fixogum Rubber Cement (Funakoshi/Marabu). The slides were then denatured on a hotplate at 74 °C for 4 min and hybridization was performed in a humidified metal box at 37 °C for 3 days. After hybridization, the slides were washed twice in 2×SSC, three times in 0.1×SSC at 62.5 °C for 5 min, and then once 0.2% Tween-20 in 4×SSC for 2min. Then, the tissue slices were blocked in 5% BSA and 0.2% Tween-20 in PBS at 37 °C for 30 min in a humidified chamber before immunostaining. The slides were incubated with mouse anti-rhodopsin antibody (1:500 dilution; Abcam, ab5417) in blocking buffer at 37 °C for 50 min, washed twice in 0.2% Tween-20 in PBS, incubated with Alexa Fluor 647-conjugated goat anti-mouse IgG antibody (1:200 dilution; Invitrogen, A-21235) in blocking buffer at 37 °C for 50 min, and washed twice in 0.2% Tween-20 in PBS. The nuclear DNA of the tissue slices was counterstained with DAPI and the slides were mounted

with Vectashield Antifade (Vector Laboratories) and sealed under cover glasses. Images of the tissue slices and cell nuclei were captured using a confocal laser scanning microscope (Carl Zeiss LSM510 meta or LSM980) equipped with a 63/1.4 Plan-Apochromat objective. For the analysis of 3D organization of the three fluorescent DNA probes with super resolution scales, several cell nuclei were scanned using the function of Airyscan 2 multiplex mode from the LSM980. The image stacks were processed with ZEN 2.3 lite and ZEN Connect to visualize the 3D reconstruction of the three fluorescent DNA probes within the cell nuclei.
